# An Improvisation in Fixation of Stamm Gastrostomy in Neonates

**Published:** 2015-01-10

**Authors:** Ab Hamid Wani, Shandip K Sinha

**Affiliations:** Department of Pediatric Surgery, Maulana Azad Medical College, and associated Lok Nayak Hospital, New Delhi, India.

**Dear Sir**

Long gap esophageal atresia with or without tracheo-esophageal fistula may require feeding gastrostomy as a part of staged reconstruction.[1] We have devised a technique to secure/anchor the feeding gastrostomy (Stamm gastrostomy) more easily and safely. Five neonates underwent feeding gastrostomy by this technique. Out of five patients, four patients had tracheo-esophageal fistula with long gap esophageal atresia and one patient had isolated esophageal atresia. No complication occurred in the post-operative period and feeding was started 12 hours after surgery. 

Under general anaesthesia, through an upper midline incision, the stomach was approached. Two purse string sutures with 4-0 vicryl were put in the middle of the stomach (knots not applied yet; ideally both sutures should start opposite to each other so that their knot should come opposite to each other for better fixation), towards lesser curvature, with the needles left intact. A small 5mm incision was made just 5cm left of the main incision at the abdominal wall, for the insertion of gastrostomy tube. The stomach was then opened under vision and silicon gastrostomy introduced under vision. The balloon was then inflated. The gastrostomy tube was then secured with tight-tying the knot of the two purse string sutures taken in the beginning of the surgery with needles still allowed to remain intact (Fig.1). A grey cannula was then inserted from abdominal wall just near the gastrostomy site to the abdomen; and the stylet was removed. Through this sheath, the two non-needle (free) ends of the thread (purse-string suture already tightened around gastrostomy tube) were brought out separately (Fig.2). The two needle ends of the purse-string suture were used to anchor the stomach with the inner abdominal wall (Fig.3). The gastrostomy tube was then fixed externally on abdominal wall with the two threads already brought out and with a separate non absorbable suture (Fig.4).

**Figure:**
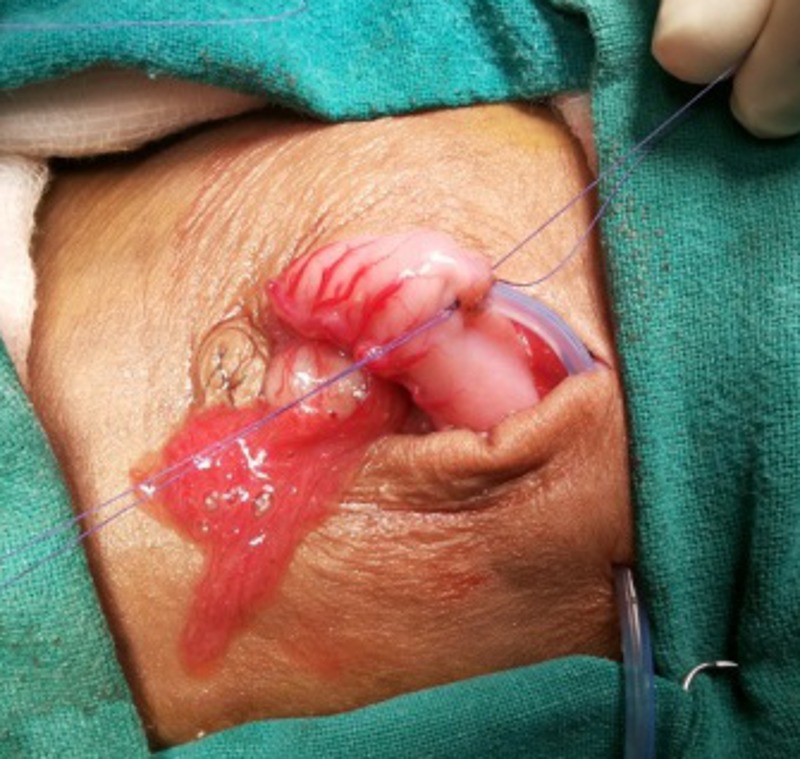
Figure 1: Tightening and tying of purse-string suture around gastrostomy tube.

**Figure:**
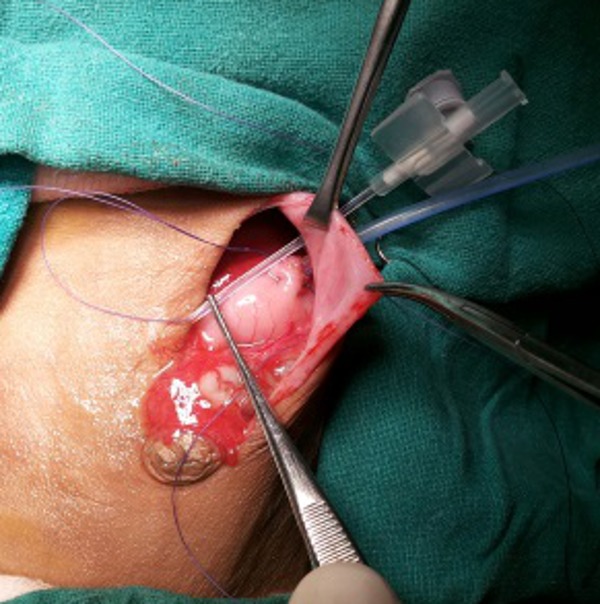
Figure 2: A cannula inserted to bring needle-free ends of purse-string sutures separately.

**Figure:**
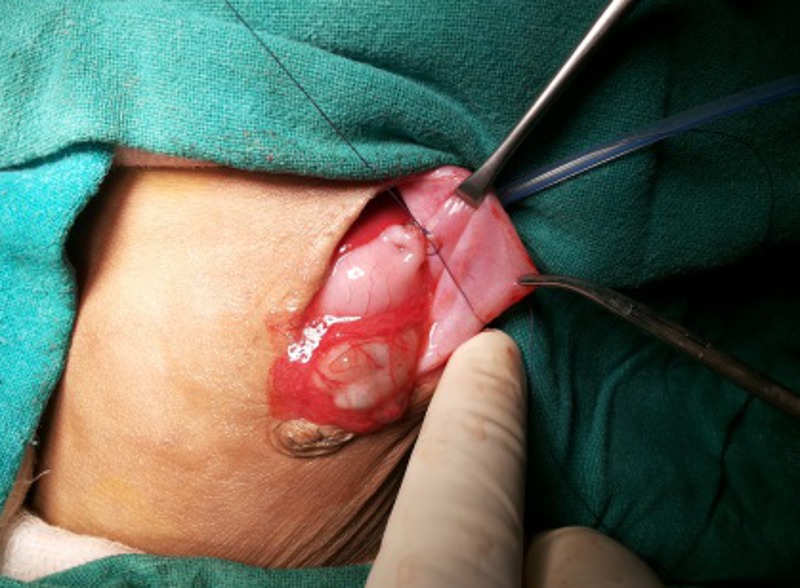
Figure 3: Needle ends used to anchor stomach with the inner abdominal wall just near gastrostomy tube insertion site.

**Figure:**
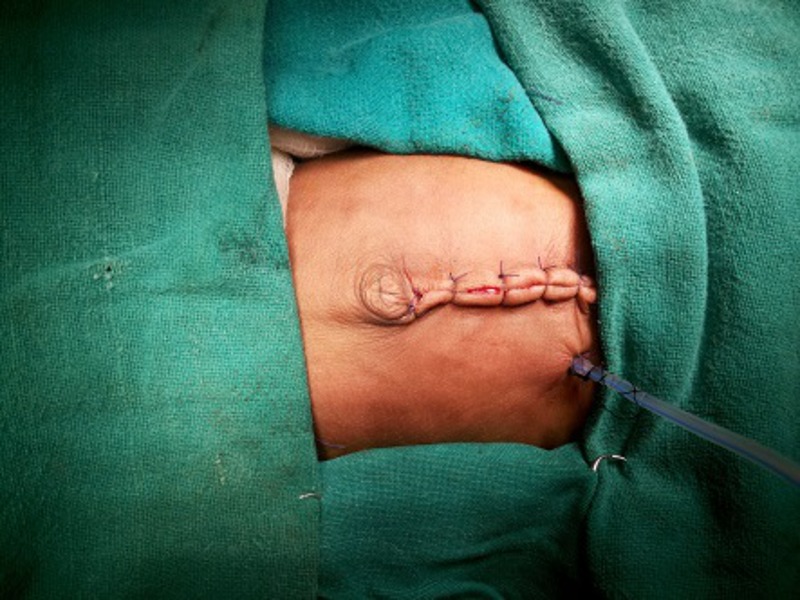
Figure 4: The gastrostomy tube was anchored externally with the needle-free ends of the thread already brought out through cannula. The gastrostomy tube was also fixed with an additional silk suture.

## Footnotes

**Source of Support:** Nil

**Conflict of Interest:** None

